# Temporal trends and social inequities in adolescent and young adult mental health disorders in Catalonia, Spain: a 2008–2022 primary care cohort study

**DOI:** 10.1186/s13034-024-00849-2

**Published:** 2024-12-18

**Authors:** Ana Lozano-Sánchez, Enric Aragonès, Tomàs López-Jiménez, Matthew Bennett, Stella Evangelidou, Esther Francisco, Myriam García, Estel Malgosa, Núria Codern-Bové, Claudia Guzmán-Molina, Constanza Jacques-Aviñó

**Affiliations:** 1 Institut Universitari d’Investigació en Atenció Primària Jordi Gol (IDIAPJGol), Barcelona, Spain; 2 Universitat Rovira i Virgili, Tarragona, Spain; 3 Atenció Primària Camp de Tarragona, Institut Català de la Salut, Tarragona, Spain; 4 Universitat Autònoma de Barcelona, Cerdanyola de Vallès, Spain; 5 Departament d’Antropologia, Filosofia i Treball Social, Universitat Rovira i Virgili, Tarragona, Spain; 6 Barcelona Institute of Global Health (ISGlobal), Barcelona, Spain; 7 Child and Adolescent Mental Health Center, Sant Joan de Déu Hospital, Cornellà, Spain; 8 Departament d’Antropologia Social i Cultural, Universitat Autònoma de Barcelona, Cerdanyola del Vallès, Spain; 9 Escola Universitària d’Infermeria i Treball Social (EUIT), Universitat Autònoma de Barcelona, Terrassa, Spain; 10 Equip d’Atenció Primària d’Artesa de Segre, Institut Català de la Salut, Artesa de Segre, Spain; 11 Unitat de Suport a la Recerca en Atenció Primària, C/ Camí de Riudoms, 53-55, 43202 Reus, Tarragona Spain

**Keywords:** Adolescents and young people, Mental health disorders, Incidence, Social inequities, Gender inequities, Cohort study

## Abstract

**Background:**

The prevalence of mental health disorders in children, teens, and young adults is rising at an alarming rate. This study aims to explore time trends in the incidence of mental disorders among young people in Catalonia, Spain from 2008 to 2022, focusing on the effects of the COVID-19 pandemic and from the perspective of social inequities.

**Methods:**

A cohort study using primary care records from the SIDIAP database was conducted. It included 2,088,641 individuals aged 10 to 24 years. We examined the incidence of depressive, anxiety, eating, and attention deficit/hyperactivity disorders, stratified by sex, age, deprivation, and nationality.

**Results:**

All disorders reflected an increasing trend throughout the study period: depressive disorders (IRR: 2.44, 95% CI: 2.31–2.59), anxiety disorders (IRR: 2.33, 95% CI: 2.27–2.39), ADHD (IRR: 2.33, 95%CI: 2.17–2.50), and eating disorders (IRR: 3.29, 95% CI: 3.01–3.59). A significant increase in incidence was observed after the outbreak of the COVID-19 pandemic. In 2022, anxiety disorders were most frequent, with an incidence rate (IR) of 2,537 per 100,000 persons-year (95% CI: 2,503–2,571). Depressive disorders followed with an IR of 471 (95% CI: 458–486), ADHD with an IR of 306 (95% CI: 295–317) and eating disorders with an IR of 249 (95% CI: 239–259). Significant associations were reported with sex, age, deprivation, and nationality.

**Conclusion:**

The incidence of all studied disorders has steadily increased, reaching unprecedented levels during the pandemic. Understanding these trends is essential for an appropriate healthcare response, while addressing the non-medical determinants, requires action across all sectors of society.

**Supplementary Information:**

The online version contains supplementary material available at 10.1186/s13034-024-00849-2.

## Background

The growing prevalence and incidence of mental health disorders in adolescents and young people is an important societal concern given its implications for the diagnosed individual, the family and society [1]. Recent Global Burden of Disease studies have revealed that mental health disorders rank among the most debilitating conditions for young people, leading to the highest number of years lived with disability with anxiety and depression topping this list [2]. In Europe, approximately 15.5% of young people are estimated to suffer from a mental disorder [3].

In high-income countries, significant socioeconomic inequalities are strongly linked to adverse mental health outcomes and health related quality of life in children and adolescents [4–7]. From social inequities perspective, structural factors as socioeconomic disadvantage, early life adversity, migratory processes, racism, discrimination due to sexual orientation or gender identity, and gender inequity contribute to worsened mental health outcomes [8]. In addition, environmental factors such as neighborhood socioeconomic status, lack of social capital, and the built environment, influence the state of mental health, especially in adolescence and young people [8]. Furthermore, increased awareness and positively evolving attitudes toward mental health, in addition to reconsidering the medicalization of feelings and behaviors once considered normal may encourage parents and adolescents to seek healthcare more readily [9, 10].

Data from various sources indicate that the burden of mental health problems among young people has been increasing over recent decades [2, 9, 11, 12], likely exacerbated by diverse societal changes and growing inequities [9, 13]. Previous studies analyzing the incidence of mental disorders in childhood and adolescence in Catalonia and the United Kingdom (UK) have reported this secular trend [14, 15]. However, these studies do not cover the timeframe that includes the impact of the COVID-19 pandemic on mental health, which is anticipated to have intensified the negative effects of socioeconomic determinants on mental health, leading to increased emotional distress and a rise in psychiatric symptoms [16, 17].

Youth and adolescence are critical periods in the development of the individuals that could be particularly vulnerable to the negative impact of social stressors [18]. These disorders can significantly affect key areas such as individual wellbeing and health, family dynamics, social interactions, and academic performance, with lasting repercussions [19]. Moreover, experiencing mental disorders during these formative years is associated with a disrupted transition to adulthood and an increased risk of mental health issues in later life [20, 21].

The World Health Organization’s (WHO) Action Plan for Mental Health 2013–2030 emphasizes the need for data on child mental health to be disaggregated by sex and age, while also considering the vulnerability of specific groups [23]. It is well established that girls are more likely than boys to report suffering from depression and anxiety, with recent reports indicating worsening internalizing symptoms among adolescent girls [22]. However, the specific reasons for this trend remain unclear, and the impact on boys is not well understood [12]. Therefore, it is essential to analyze the specific effects of sex on the incidence of mental disorders in adolescents and young people. This study provides valuable insights for designing public policies that effectively address the mental health needs of adolescents and young adults, aligning with the goals of the WHO Comprehensive Mental Health Action Plan 2013–2030. [23].

The objective of this study was to explore temporal trends in the incidence of several mental disorders in adolescents and young adults in Catalonia, Spain, according to demographic characteristics and social inequities (sex, age, deprivation and nationality) during the period 2008–2022, with a particular focus on the impact of the COVID-19 pandemic on these outcomes.

## Methods

### Study design, setting and data source

We carried out a cohort study using primary care records spanning from January 1, 2008 to December 31, 2022 in Catalonia, Spain. We utilized individual-level data extracted from electronic health records from 328 primary care centers managed by the Catalan Institute of Health. Data from these records are systematically compiled to make up the Information System for Research in Primary Care (SIDIAP) database. Since its establishment in 2006, SIDIAP has gathered records for over 8 million individuals. In June 2021, with 5.8 million people represented in the database, corresponding to approximately 75% of the total resident. SIDIAP is representative of the general population of Catalonia in terms of age, sex, and geographic distribution [24]. Psychiatric diagnoses recorded in primary care medical records include those made within primary care as well as diagnoses from other healthcare levels, due to the high degree of integration between primary care and psychiatry within the Catalan healthcare system [25].

The WHO declared COVID-19 a pandemic in March 2020 and officially declared its end in March 2023 [26, 27]. For the purposes of our study, we defined the pandemic period as encompassing the years 2020, 2021, and 2022.

### Study participants

An open cohort was established, allowing for the inclusion or exclusion of participants over time. All individuals registered in the SIDIAP aged 10 to 24 years old at any point during the study period were considered for inclusion. Individuals were enrolled in the cohort either on the study’s start date (1 January 2008) or when they reached the minimum age for inclusion (10 years old) after the start of the study. Individuals were followed until diagnosis of a mental health disorder within one of the defined categories, reaching the maximum age (25 years old), transferring out of SIDIAP, death, or the end of the study period.

### Variables

#### Outcomes

The study focused on the incidence of specific mental health disorders included in the following categories: (1) depressive disorders (F30-F39), (2) anxiety disorders (F40-F44), (3) eating disorders (F50), and (4) attention-deficit/hyperactivity disorder (ADHD) (F90). Conditions were identified based on the International Statistical Classification of Diseases and Related Health Problems, 10th Revision (ICD-10) codes [28]. The first recorded code for each outcome category was considered an incident episode. Focusing on adolescent depression, anxiety, ADHD, and eating disorders is a strategic choice due to their high prevalence, public health impact, and emergence during adolescence—a critical developmental stage [2, 3]. Moreover, these conditions often have long-term consequences into adulthood [20]. Their distinct yet interconnected nature highlights overlapping risk factors, and their inclusion in a large-scale epidemiologic study ensures robust trend analysis. The growing influence of social factors on these conditions [13] aligns with the study aims.

#### Covariates

We used sociodemographic data recorded in SIDIAP, including sex (male or female), age groups (10 to 14 years, 15 to 19 years, and 20 to 24 years, respectively early adolescence, late adolescence and young adulthood) [29], and nationality (Spain, other European countries, Northern America, Central and South America, Africa, and Asia/Oceania). To assess socioeconomic status, we employed the MEDEA Deprivation Index (Mortality in Small Spanish Areas and Socioeconomic and Environmental Inequalities), which was assigned to each census area. This index is derived from several indicators: proportion of manual workers, temporary salaried workers, unemployment rates, insufficient education, and percentage of primary residences without internet access. This index is applicable to urban areas. The deprivation index is divided into quintiles, with the first and fifth quintiles denoting the least and most deprived areas, respectively [30].

### Statistical methods

We conducted a descriptive analysis to describe the sociodemographic characteristics of the population. Continuous variables were summarized using the median and Interquartile Range (IQR), while categorical variables were presented as absolute sums and percentages.

The annual incidence of depressive disorders, anxiety disorders, eating disorders, and ADHD was calculated separately. To do so, the number of individuals aged 10–24 years diagnosed with each mental disorder (on or before January 1 of each year) was divided by the total number of individuals aged 10–24 years on January 1 of each calendar year of study.

For incidence calculations, individuals without a prior history of the specific mental health disorder before January 1, 2008, contributed person-time starting from the date they became eligible for the study (aged 10 years at any time during follow-up) until the first occurrence of, their 25th birthday, death, transfer out of SIDIAP, or the study’s conclusion on December 31, 2022.

Annual Incidence Rates (IRs) of mental disorder diagnoses were calculated from 2008 to 2022 by dividing the number of new cases by 100,000 person-years at risk. These IRs were calculated using the traditional formula (i.e., no models were applied to calculate IRs). We only took into account first-ever diagnoses. All analyses were stratified IRs by sex, age groups (calculated annually), nationality, and deprivation index quintiles. Following a thorough visual examination of incidence curves over the study period, several significant trends emerged spanning from 2008 to 2022, including growth, decline, and stabilization patterns. To quantify these trends, we calculated Incidence Rate Ratios (IRRs) to compare incidence rates between the start and end of each defined period. IRRs were estimated using Poisson regression, allowing us to calculate 95% confidence intervals and evaluate statistical differences in incidence rates across periods and sociodemographic groups. This distinction between the traditional calculation of IRs and the regression-based estimation of IRRs ensures appropriate analysis for each purpose.

Regarding the MEDEA deprivation index, 13.5% of individuals in rural areas–where MEDEA is not applicable– and 16.9% with unknown residence were excluded from the analysis (completed-cases analysis).

The analyses were conducted using SPSS 25.0 (SPSS Inc., Armonk, NY: IBM Corp) and R version 4.3.2.

## Results

We utilized data from a total of 2,088,641 eligible individuals, accumulating 13,136,826 person-years of observation [Fig. S1, see Additional file 1].

Among the participants enrolled, 1,022,600 (49.0%) were female. Upon cohort entry, the median age of the cohort was 12.0 years, with an interquartile range spanning from 10.0 to 19.2 years. Additional sociodemographic data are available in Table S1 [see Additional file 1].

Anxiety disorders were the most commonly diagnosed mental illnesses among the studied ones, with a IR (incidence rate) in 2022 of 2537 per 100,000 persons-year (95%CI: 2503–2571). The other disorders studied presented lower incidence: depressive disorders IR: 471 (95%CI: 458–486), ADHD IR: 306 (95%CI: 295–317) and eating disorders IR: 249 (95% CI: 239–259). Annual incidence rates over the entire study period for all the mental health disorders of study stratified by sociodemographic characteristics can be found in Tables S2, S3, S4, and S5 [see Additional file 1].

### Time trends in the incidence of mental disorders

**Fig. 1:**
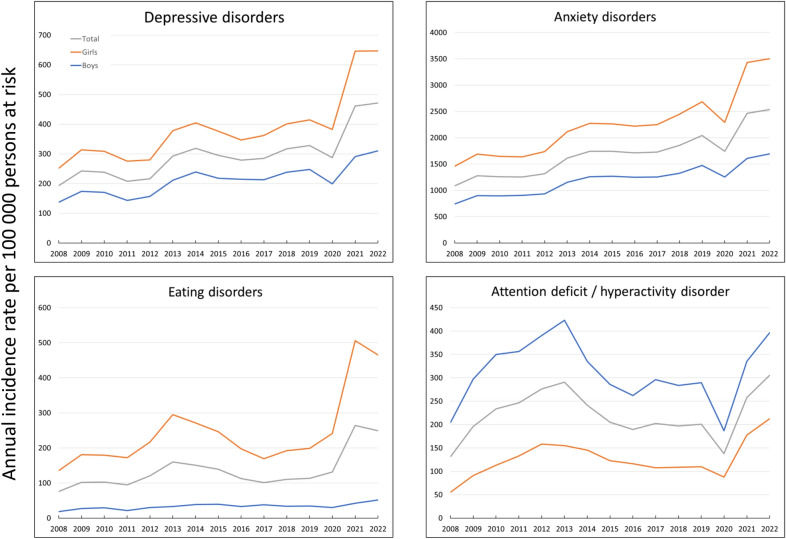
Temporal trends in annual incidence rates of mental health disorders by age groups, 2008–2022

We have observed a fluctuating yet overall increasing trend in the IR of depressive disorders (IRR: 2.44, 95% CI: 2.31–2.59) and anxiety disorders (IRR: 2.33; 95% CI: 2.27–2.39), throughout the entire study period from 2008 to 2022. Notably, both disorders experienced a decline in incidence in 2020 compared to 2019, followed by a significant upsurge in incidence rates during the period from 2020 to 2022. In this period (2020 to 2022), for depressive disorders we found IRR: 1.64 (95% CI: 1.56–1.72); and for anxiety disorders IRR: 1.46 (95% CI: 1.43–1.49) (Fig. 1, Table 1).

**Table 1:** IRR for mental health disorders in people 10–24 years old by sex, age, deprivation and nationality

	IRR 2022 vs. 2008 (the entire study period)		IRR 2013 vs. 2011 (peak period of economic crisis)		IRR 2020 vs. 2019 (lockdown/restriction of access to health services)		IRR 2022 vs. 2020 (pandemic period)	
Depressive disorders	2.44 (2.31–2.59)		1.41 (1.32–1.50)		0.88 (0.83–0.92)		1.64 (1.56–1.72)	
Anxiety disorders	2.33 (2.27–2.39)		1.28 (1.25–1.32)		0.85 (0.83–0.87)		1.46 (1.43–1.49)	
Eating disorders	3.29 (3.01–3.59)		1.68 (1.54–1.84)		1.16 (1.06–1.26)		1.90 (1.77–2.04)	
ADHD	2.33 (2.17–2.50)		1.18 (1.11–1.25)		0.69 (0.64–0.74)		2.22 (2.07–2.37)	

	IRR (95% CI) by sex, year 2022		IRR (95% CI) by age group, year 2022		IRR (95% CI) by deprivation quintiles, year 2022		IRR (95% CI) by nationality, year 2022	
Depressive disorders	Boys	1.00	10–14 y/o	1.00	Q5 (most deprived)	1.00	Spain	1.00
	Girls	2.08 (1.96–2.22)	15–18 y/o	1.25 (1.15–1.36)	Q4	0.93 (0.84–1.04)	Americas	1.50 (1.35–1.68)
			19–24 y/o	1.52 (1.41–1.63)	Q3	0.98 (0.88–1.09)	Africa	0.56 (0.46–0.67)
					Q2	0.98 (0.88–1.09)	Asia and Oceania	0.45 (0.35–0.58)
					Q1 (least deprived)	0.87 (0.79–0.97)	Other European	0.90 (0.76–1.07)
Anxiety disorders	Boys	1.00	10–14 y/o	1.00	Q5 (most deprived)	1.00	Spain	1.00
	Girls	2.07 (2.01–2.13)	15–18 y/o	1.59 (1.53–1.66)	Q4	1.00 (0.96–1.05)	Americas	1.59 (1.52–1.67)
			19–24 y/o	2.25 (2.18–2.33)	Q3	0.96 (0.92–1.01)	Africa	0.74 (0.69–0.80)
					Q2	0.87 (0.83–0.91)	Asia and Oceania	0.43 (0.38–0.48)
					Q1 (least deprived)	0.70 (0.67–0.73)	Other European	1.05 (0.97–1.12)
Eating disorders	Boys	1.00	10–14 y/o	1.00	Q5 (most deprived)	1.00	Spain	1.00
	Girls	8.96 (7.87–10.21)	15–18 y/o	1.16 (1.06–1.28)	Q4	1.15 (0.99–1.33)	Americas	0.84 (0.69–1.01)
			19–24 y/o	0.41 (0.37–0.46)	Q3	1.16 (1.00–1.35)	Africa	0.26 (0.18–0.37)
					Q2	1.23 (1.06–1.43)	Asia and Oceania	0.25 (0.16–0.39)
					Q1 (least deprived)	1.09 (0.94–1.27)	Other European	0.80 (0.63–1.01)
Attention deficit and hyperactivity disorder	Boys	1.00	10–14 y/o	1.00	Q5 (most deprived)	1.00	Spain	1.00
	Girls	0.54 (0.50–0.58)	15–18 y/o	0.39 (0.36–0.43)	Q4	1.07 (0.92–1.23)	Americas	0.80 (0.67–0.95)
			19–24 y/o	0.16 (0.14–0.18)	Q3	1.01 (0.87–1.17)	Africa	0.37 (0.29–0.49)
					Q2	1.08 (0.94–1.25)	Asia and Oceania	0.16 (0.10–0.27)
					Q1 (least deprived)	1.38 (1.20–1.60)	Other European	0.68 (0.54–0.86)

(1) The analyzed periods were chosen following a visual examination of the graphs to capture significant variations in incidence rates over the follow-up. (2) For comparisons between the different subgroups defined by covariates (sex, age, deprivation, and nationality), the IRRs are shown for the year 2022, as it represents the most recent observation

Regarding eating disorders, the IRR between 2022 and 2008 was 3.29 (95% CI: 3.01–3.59), increasing especially after 2020. In 2021, there was a notable increase in its incidence (IRR between 2021 and 2020: 2.75, 95% CI: 2.57–2.95), which then stabilized in 2022. This phenomenon was particularly evident in girls (Fig. 1, Table 1).

ADHD presented irregular trends from 2008 to 2022, with peak incidence observed in 2013 (IRR between 2013 and 2008: 2.21, 95% CI: 2.06–2.38). In 2020, there was a notable decrease compared to 2019 (IRR: 0.69, 95% CI: 0.64–0.74), followed by a sharp increase in the period from 2020 to 2022 (IRR: 2.22, 95% CI: 2.07–2.37) (Fig. 1, Table 1).

### Sex

In terms of sex-specific trends, the incidence rates of depressive and anxiety disorders exhibited parallel trends over the entire study duration. However, the incidence in girls is consistently double that of boys. For example, in 2022 the IRR between girls and boys for depressive disorders was 2.08 (95% CI: 1.96–2.22), and for anxiety disorders was 2.07 (95% CI: 2.01–2.13) (Fig. 1, Table 1).

In the case of eating disorders, incidence rates were manifestly higher in girls compared to boys (in 2022 the IRR between girls and boys was 8.96, 95% IC: 7.87–10.21).

Finally, for ADHD, we observed parallel trends between both sexes, with the highest incidence consistently observed in boys. For example, in 2022, the IRR between girls and boys was 0.54 (95% CI: 0.50–0.58) (Fig. 1, Table 1).

### Age

**Fig. 2:**
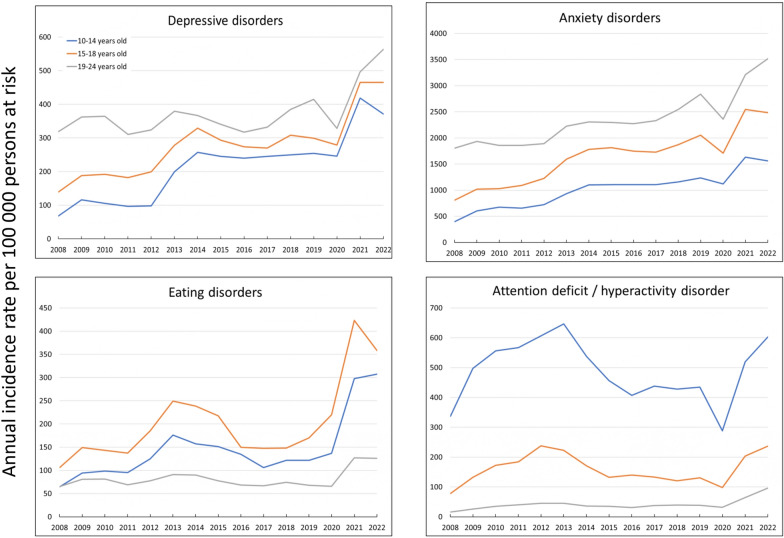
Temporal trends in annual incidence rates of mental health disorders by age groups, 2008–2022

In the growing secular trend in the incidence rates of depressive and anxiety disorders, the trajectories in the three age groups were generally the same. Notably, the incidence among the youngest age group remains consistently lower than that of the other age groups. For example, in 2022, the IRR for depressive disorders between the 19–24 year-old age group and 10–14 year-old age group was 1.52 (95% IC: 1.41–1.63) and for anxiety disorders the IRR was 2.25 (95% CI: 2.18–2.33) (Fig. 2, Table 1).

In the case of eating disorders, the highest incidence rates were observed among the 15–18 year-old age group, followed by the 10–14 year-old age group. While these two groups present similar time trend evolutions, the 19–24 year-old age group presents lower and relatively stable incidence levels throughout the entire study period. However, IRs double across all age groups experience after 2020 (Fig. 2, Table 1).

Regarding ADHD, the highest incidence was noted in the 10–14 year-old age group, with a notable increase from 2008 to 2013 (IRR: 1.92, 95% IC: 1.77–2.08), followed by a subsequent decrease. After a sharp decline in 2020, incidence rebounded until 2022 (between 2022 and 2020, in the 10–14 year-old age group IRR: 2.09, 95% CI: 1.93–2.27). The 15–18 year-old age group demonstrates similar trends, albeit at a lower level, while the 19–24 year-old age group maintains consistently low levels of incidence during the study period, with an upswing from 2020 onwards (Fig. 2, Table 1).

### Deprivation

**Fig. 3:**
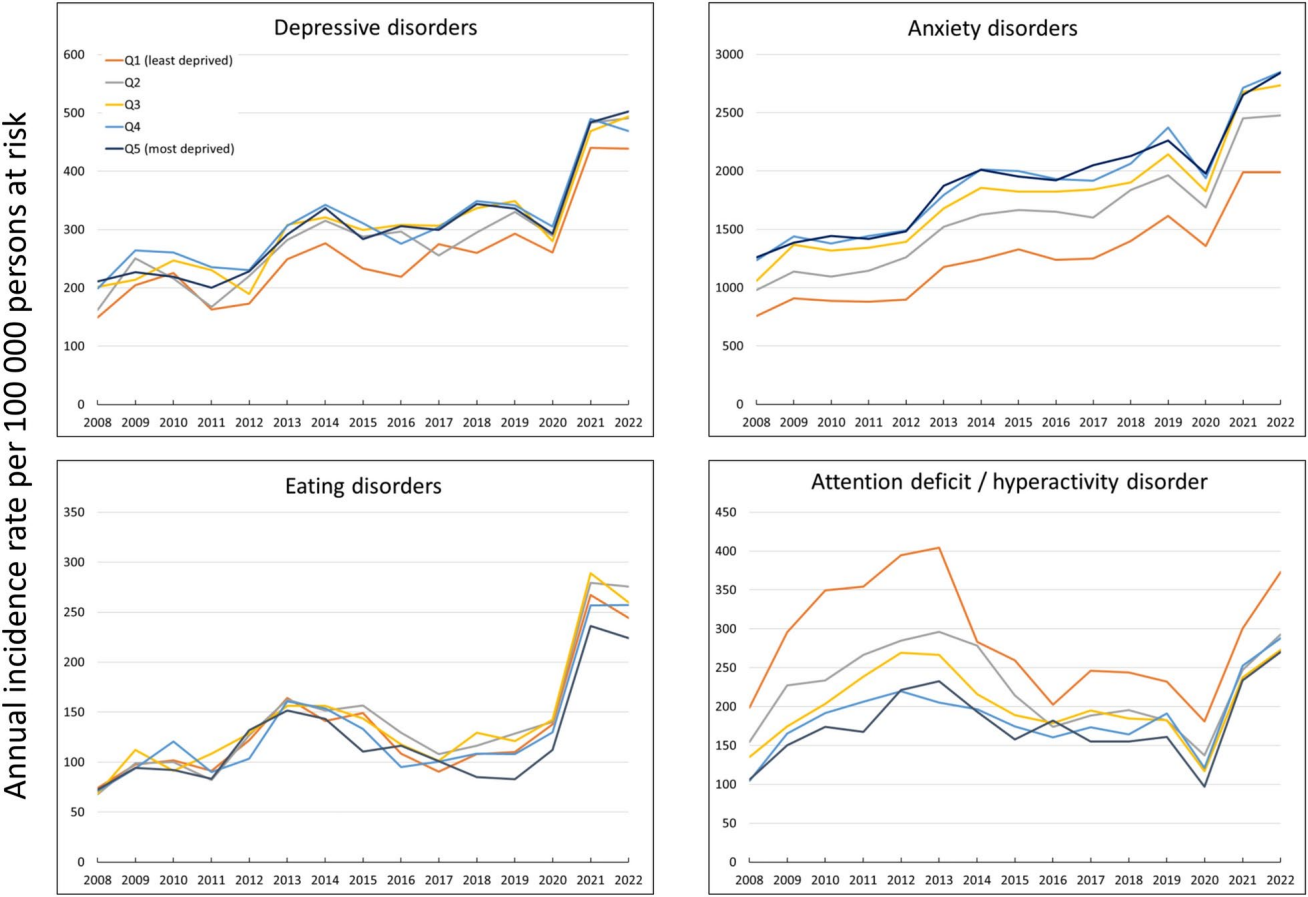
Temporal trends in annual incidence rates of mental health disorders by deprivation quintiles, 2008–2022

In both depressive and anxiety disorders, incidence curves by deprivation levels exhibit similar trends throughout the study period and present some intersections and overlaps. The least deprived group generally presents lower IRs compared to the other groups, particularly evident in anxiety disorders. For example, in 2022, the IRR between the least and the most deprived groups was 0.87 (95% CI: 0.79–0.97) for depressive disorders, and 0.70 (95% CI: 0.67–0.73) for anxiety disorders (Fig. 3, Table 1).

Similarly, in eating disorders, the incidence curves overlapped between different deprivation levels. However, in 2022, the incidence among the most deprived group was lower than that of the other groups (Fig. 3, Table 1).

In ADHD, the curves of the deprivation index quintiles remain parallel throughout the study period, showing an inverse correlation between degree of deprivation and incidence: the lower the deprivation level, the higher the incidence of ADHD. In 2022, the IRR between the most and the least deprivation level was 1.38 (95% CI: 1.20–1.60) (Fig. 3, Table 1).

### Nationality

**Fig. 4:**
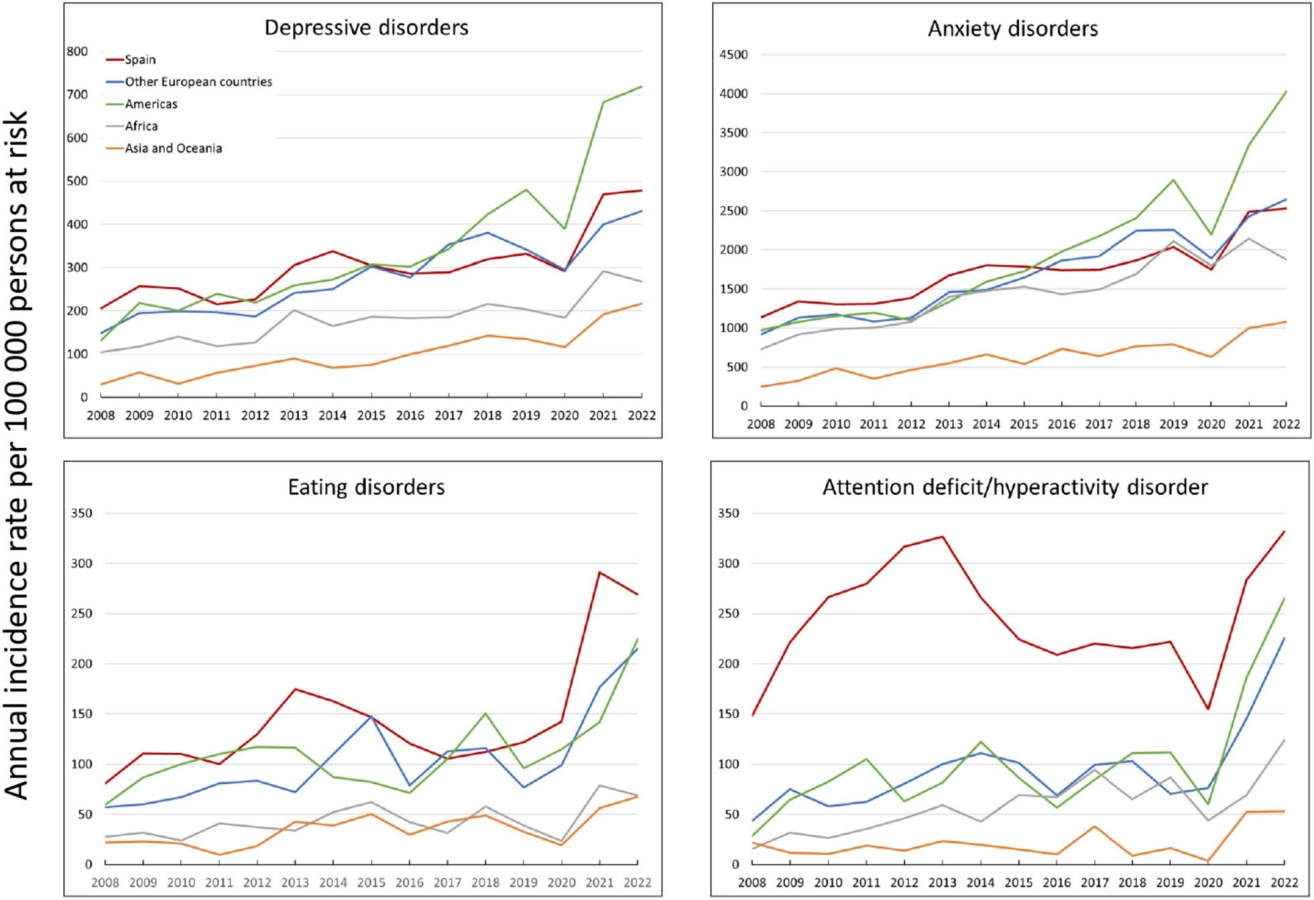
Temporal trends in annual incidence rates of mental health disorders by nationality, 2008–2022

For all the disorders, the incidence curves for non-Spanish nationals generally remain at lower levels compared to those for individuals with Spanish nationality. Notably, incidence rates among individuals with North and South America nationality increase from 2015 onwards, exceeding the incidence rates of those with Spanish nationality. In 2022, the IRR between North and South American vs. Spanish nationality groups was 1.50 (95% CI: 1.35–1.68) for depressive disorders, and 1.59 (95% CI: 1.52–1.67) for anxiety disorders (Fig. 4, Table 1).

## Discussion

### Overview of findings

This study highlights a significant overall increase in the incidence of all mental health diagnoses during the COVID-19 pandemic, with anxiety disorders being the most common. Notably, the incidence of all studied mental health disorders was higher in girls compared to boys, with the exception of ADHD. Additionally, there was an elevated incidence of depression and anxiety diagnoses among individuals aged 19–24 years, while ADHD was more frequent in the younger cohort aged 10–14 years. Depressive and anxiety disorders were more common among individuals from the most deprived areas, whereas eating disorders and ADHD were more frequent among those from less deprived areas. Furthermore, the incidence of mental health disorders was generally lower among individuals of non-Spanish nationality compared to Spanish nationals.

### Incidence topics

Anxiety was the most frequent diagnosis during our study period, with incidences 5-times that of depression or about 10-times that of ADHD or eating disorders. While the higher incidence of anxiety disorders compared to other disorders is expected, it appears excessively disproportionate considering epidemiological data [31]. Anxiety symptoms can be part of the clinical expression of other mental disorders or can precede the manifestation of other symptoms or mental health disorders [32]. Therefore, a significant percentage of the diagnoses of anxiety registered in primary care records may be an indicator of nonspecific emotional distress or preliminary states of other disorders.

We compared incidence our rates of psychiatric disorders with data from Cybulski in the UK [15] for 2018 focusing on the age range of 10 to 19 years, selected due to data availability. Anxiety disorder rates were similar: 203 and 106 cases per 10,000 for girls and boys in our cohort, versus 199 and 92 in the UK. However, depression rates were over 4-times lower in our cohort (36 and 22 per 10,000 for girls and boys) compared to much higher UK rates (176 and 98 per 10,000). Eating disorder rates were comparable, with 23 cases per 10,000 among girls in our cohort and 17 in the UK. ADHD rates, however, were higher in our cohort (40 cases per 10,000 for boys) compared to 19 in the UK. These discrepancies, particularly the pronounced differences in depressive disorders and ADHD, raise questions about whether they reflect true variations in the incidence of psychiatric disorders or, alternatively, result from differences in recording practices across healthcare systems.

### Temporal trends

#### Secular trends (2008–2022)

We noticed a consistent increase in incident depressive and anxiety disorders from 2008 to 2022, which aligns with the findings of longitudinal studies conducted in the UK [15] as well as a previous study carried out in Catalonia that used a different database [14]. Recent reviews corroborate the ongoing secular increase in the incidence of mental health disorders in adolescents, particularly in high-income countries, which is plateauing globally [33].

#### Impact of the economic crisis (2011–2013)

We observed increases in the incidence of all disorders in the years 2011–2013. This phenomenon, which has already been documented both in Catalonia [14] and in the UK [15], may be related to the impact of the economic crisis that began in 2008 [34]. The crisis reached its peak social impact in Spain in 2011 and continued in the following years due to austerity policies affecting social welfare, health, and education. [35].

#### Impact of the COVID-19 pandemic (2020–2022)

Nevertheless, in this study a sharp increase in incidence of studied mental health disorders was noted following the onset of the COVID-19 pandemic. In 2020, a temporary dip in the incidence curves of anxiety, depression, and ADHD is observed. This finding aligns with other epidemiological studies on mental health disorders [36, 37] and can be attributed to the lockdown and restrictions on access to healthcare services during the early months of the COVID-19 pandemic [38, 39]. Longitudinal studies that calculated the incidence rates of mental health disorders over shorter periods (monthly or bimonthly) show a notable decrease in new recorded diagnoses in the initial months after the outbreak, with a gradual return thereafter to expected values [36, 37]. Following this decline, there has been a dramatic increase in all studied disorders until 2022. This surge reflects the adverse effects on adolescent and youth mental health resulting from the pandemic, as well as that of the lockdown and social distancing measures that were implemented. These impacts have been observed using different methodologies in many countries around the world [16–18]. The sharp rise in mental health diagnoses during the pandemic period may also reflect heightened societal and healthcare system awareness of mental health. Factors such as reduced stigma, improved mental health literacy, and greater prioritization of mental health care likely contributed to this trend [10].

### Sociodemographic patterns

#### Gender disparities

The results of this study reveal a worsened state of mental health among girls, with a higher incidence in all disorders except ADHD. This disparity may be partly attributed to a social system where sexism and other forms of violence against women negatively impact mental health [8,[40]. Additionally, the greater prevalence of diagnoses in girls could be linked to the gender socialization process, which may enhance girls’ ability to express psychological discomfort [40]. The results suggest that psychological distress manifests differently by gender, with girls more likely to express it emotionally, while boys tend to express it behaviorally [40]. This would correspond to the higher incidence in girls of depressive, anxiety, and eating disorders, and in boys of ADHD. ADHD incidence is consistently higher in boys than in girls throughout the study period, which could be attributed to sex bias in its clinical diagnosis process: girls with ADHD may be more easily overlooked due to a higher symptom threshold requirement for seeking help and diagnosis [41].

For eating disorders, the results reveal higher incidence in girls than in boys, in line with many other studies which indicate a greater presence of these disorders in girls [42]. This may be due to gender norms related to body image and weight [43] though other research suggests these disorders express different cultural norms, values, and conflicts influenced by gender and the sociocultural context [44].

#### Age-related trends

According to age, our findings are consistent with the idea that internalized symptoms are more prevalent in older adolescents and young adults, whereas externalized symptoms are more prevalent in children and adolescents [45]. Thus, the 15–18 and 19–24 age groups would score higher for depressive and anxiety disorders, while the 10 -14 age group would score higher for ADHD. Age is a determining factor in the incidence of ADHD, displaying a gradient: the incidence is higher in the younger age group (10–14 years) while new ADHD diagnoses are infrequent in the age group over 18 years old. A limitation of our data is that we did not examine individuals under the age of 10, when, according to epidemiological data, the peak incidence by age is precisely around 7–9 years old [46].

#### Socioeconomic and nationality factors

Our study also observed a noteworthy relationship between socioeconomic deprivation and the incidence of ADHD and eating disorders, which diverges from patterns seen in other disorders. While depressive and anxiety disorders show higher incidence among individuals in the most deprived groups, ADHD presents higher incidence among individuals in the least deprived groups. This contradicts findings where ADHD symptoms, diagnoses, and treatments were more frequent among individuals in most deprived groups [46–48]. Other studies reveal the mechanisms of more frequent ADHD diagnoses among individuals of high socioeconomic status, including heightened awareness among parents and teachers, greater academic performance expectations, higher health literacy, and improved access to healthcare [49, 50]. ADHD is a neurodevelopmental disorder with a well-established genetic and biological basis [51]. However, social and structural inequalities, such as those related to sex, socioeconomic status, or nationality—factors assessed in this study—,differentially affect healthcare access and symptom interpretation, often contributing to disparities in diagnosis and recorded incidence [52]. Moreover, the DSM-5 expanded the criteria for ADHD and lowered the diagnostic threshold, which may partially explain the peak in diagnoses around 2013 [51].

Our finding of a lower incidence of eating disorders in the most deprived stratum seems to suggest that these conditions predominantly occur in less deprived groups. However, it is important to acknowledge that our data reflect recorded diagnoses rather than actual incidence. Stereotypes, such as the misconception of eating disorders as “diseases of affluence” may contribute to diagnostic gaps, particularly among individuals from more deprived backgrounds [53]. Structural barriers, including limited access to mental health services, further exacerbate these disparities. Additionally, clinicians may be less likely to identify eating disorder symptoms in individuals who do not fit stereotypical profiles, perpetuating underdiagnosis and systemic inequities [54, 55]. From an intersectional perspective, the interaction of socioeconomic deprivation with other social identities, such as race and ethnicity, has been found to amplify these inequalities [56]. These intersecting axes of disadvantage likely influence our finding of a higher incidence of diagnosed eating disorders among individuals of Spanish nationality compared to those of foreign nationalities.

Our study surprisingly revealed lower incidence of the studied mental health disorders among non-Spanish nationals compared to those with Spanish nationality. Despite universal coverage offered by the Spanish public healthcare system, barriers related to language and culture, lack of familiarity with rights, gaps in health literacy, limited knowledge of the health system, discrimination, and socioeconomic inequity lead to differential treatment within the healthcare system [57]. However, those of American nationality have shown a strong and sustained increase in the incidence of depression and anxiety since 2015, accelerating from the outbreak of the COVID-19 pandemic, resulting in their rates surpassing those of individuals with Spanish nationality. The specific factors behind these differential incidence trends merit further research.

### Implications and broader context

There is a significant issue of psychological distress and adverse mental health among adolescents and young people, which have been exacerbated by the COVID-19 pandemic, though they preceded it [33]. Structural factors, discrimination and violence are risk factors, as they are different expressions of psychological distress between genders, age groups and socioeconomic status [8, 40, 44, 45]. Parental psychopathology, living in an urban context, excessive use of the internet and social networks, among others, have been identified as risk factors in previous research [58, 59], mediated by additional factors that facilitate or promote seeking help in the healthcare system [60]. Understanding this phenomenon is necessary for an appropriate response from the healthcare system and, since many relevant determinants are not healthcare-related, the response must also be rooted in societal change [61].

### Limitations and strengths

This study has limitations that must be considered when interpreting the results. Firstly, the data pertain to diagnoses recorded in primary care medical records, not the actual incidence of mental disorders in the population. Underdiagnosis or overdiagnosis can contribute to discrepancies between recorded diagnoses and the actual presence of disorders in the population [50, 62]. Additionally, increased knowledge and awareness among physicians and pediatricians about mental disorders, decreased stigma, and social trends regarding mental health, can facilitate increased help-seeking behavior and mental health diagnoses [10]. A related potential source of bias in our database is that diagnoses may be more common in severe cases because these individuals could be more likely to seek medical care and easily detected by doctors [63]. Secondly, diagnoses given in specialized psychiatry settings may not be adequately recorded in primary care medical records. However, within the Catalan public health system, primary and specialty care are integrated, reducing underreporting [25]. The management of chronic diagnoses initiated in other levels of care,–including private healthcare–, usually occur in primary care [64], reducing the probability of undocumented diagnostic information. Thirdly, for the gender-based analysis, we used the variable “sex” as a proxy. However, we acknowledge that gender is a far more complex construct that cannot be limited to a simple binary biological category [65]. Fourthly, the MEDEA variable is constructed at the ecological level based on census areas [30]. While this variable is a useful proxy for individual socioeconomic deprivation, its ecological nature introduces potential dependencies between individuals within the same area. A multilevel analysis could account for such dependencies, but this approach was not undertaken, as our primary objective was to stratify results by MEDEA quintiles rather than model hierarchical relationships. Future studies exploring contextual effects at the area level may consider incorporating multilevel modeling to address this issue. And finally, the variable “nationality” must be interpreted with caution since we lack other data regarding origin, years of residence in the country, level of integration, or other relevant factors [66].

The strengths of the study lie in the sample size and the use of real-world origin of the data. Our study reports broadly representative data, including about two million adolescents and young adults seen in primary care in Catalonia. The SIDIAP database has been proven to be a valid and useful tool for research purposes [24]. Moreover, the extended study period allows us to analyze and interpret the situation within the context of the COVID-19 pandemic from a temporal perspective, including the secular evolution of the outcomes of interest since 2008. Additionally, our study encompasses the entire period of the COVID-19 pandemic, from its onset in early 2020 to the end of 2022, shortly before the formal declaration of the end of the pandemic by the WHO in March 2023 [26, 27].

## Conclusion

In this study, we have observed a secular increase in the incidence of the studied disorders, a trend that has accelerated with the onset of the COVID-19 pandemic, pushing incidence rates to unprecedented levels. It remains uncertain whether these incidence rates will continue to rise, stabilize, or return to pre-pandemic levels. Future longitudinal studies will be necessary to monitor this phenomenon. Our findings indicate that mental health disorders were reported predominantly in girls, 15–18 and 19–24 year olds, those of most deprived areas, and Spanish nationality.

This study leaves questions unanswered, such as the relationship between migratory status and incidence, the response of the healthcare system, gender biases in the actual incidence and clinical management of disorders, and the role of socioeconomic inequities, which have been studied here in a generic way using an ecological indicator but deserve deeper investigation. In addition to longitudinal studies, it is crucial to explore these phenomena further through qualitative studies involving the active participation of those affected. This approach will help provide a comprehensive understanding of the data presented in this article.

## Supplementary Information

Supplementary file1 (DOCX 67 kb)

## Data Availability

Data cannot be shared publicly because of ethical restrictions. The Ethical Committee does not allow us to share the data publicly as our data contain sensitive personal information and cannot be fully anonymized. Data are available from the Research Ethics Committee of the Institut de Recerca en Atenció Primària Jordi Gol i Gurina (IDIAPJGol) (contact via cei@idiapjgol.info) for researchers who meet the criteria for access to confidential data.

## Abbreviations

IR: Incidence rate
ADHD: Attention deficit/hyperactivity disorder
UK: United Kingdom
WHO: World Health Organization
SIDIAP: Information system for research in primary care
ICD-10: International statistical classification of diseases and related health problems, 10th revision
MEDEA: Mortality in small Spanish areas and socioeconomic and environmental inequalities deprivation index
IQR: Interquartile range
IRs: Annual incidence rates
