# Supplementary material for: Temporal trends and social inequities in adolescent and young adult mental health disorders in Catalonia, Spain: a 2008–2022 primary care cohort study
Source: Child Adolesc Psychiatry Ment Health. 2024 Dec 18;18:159. doi: 10.1186/s13034-024-00849-2 (PMC11657204; doi:10.1186/s13034-024-00849-2)
Supplement: Supplementary file 1 — Supplementary file1 (DOCX 67 kb) [file 13034_2024_849_MOESM1_ESM.docx]

**ADDITIONAL FILE 1**

**Supplementary Material**

**Temporal trends and social inequities in adolescent and young adult mental health disorders in Catalonia, Spain: a 2008-2022 primary care cohort study.**

*Child and Adolescent Psychiatry and Mental Health*

Study population at the end

(31 December 2022)

N= 953,730

Individuals who leave the cohort throughout the study period, n= 1,134,911

- By transfer, n= 231,223
- By death, n= 2,072
- For turning 25 y/o, n= 901,523
- Not at risk*, n= 93

Eligible study population at the beginning (01 January 2008)

N=768,943

Excluded n=582,635

- Less than 10 y/o, n= 582,634
- Not at risk*, n= 1

Population up to 24 y/o in SIDIAP database on 01 January 2008

N=1,351,578

Individuals who join the cohort throughout the study period, n=1,319,698

- By transfer, n= 408,154
- For turning 10 y/o, n= 911,544

* i.e., with previous diagnoses of all the disorders considered in the study

**Figure S1. Flowchart with the entry and exit of participants within the cohort over the course of the study period.**

**Table S1. Sociodemographic characteristics of the study cohort**

|  | **Total** | **With depressive disorders** | **With anxiety disorders** | **With eating disorders** | **With ADHD** |
| --- | --- | --- | --- | --- | --- |
|  | (N=2,088,641) | (n=38,587) | (n=204,776) | (n=17,781) | (n=28,124) |
| Sex |  |  |  |  |  |
| Girls, n (%) | 1,022,600 (49.0%) | 24,394 (63.2%) | 128,974 (63.0%) | 15,512 (87.2%) | 7,962 (28.3%) |
| Boys, n (%) | 1,066,041 (51.0%) | 14,193 (36.8%) | 75,802 (37.0%) | 2,269 (12.8%) | 20,162 (71.7%) |
| Age, median (IQR) | 12.0 (10.0-19.2) | 10.7 (10.0-16.3) | 11.1 (10.0-16.4) | 10.0 (10.0-12.8) | 10.0 (10.0-12.3) |
| Age group, n (%) |  |  |  |  |  |
| 10-14 y/o | 1,242,638 (59.5%) | 26,904 (69.7%) | 140,498 (68.6%) | 14,948 (84.1%) | 26,627 (94.7%) |
| 15-18 y/o | 303,221 (14.5%) | 6,109 (15.8%) | 34,457 (16.8%) | 1,800 (10.1%) | 1115 (4%) |
| 19-24 y/o | 542,782 (26.0%) | 5,574 (14.4%) | 29,821 (14.6%) | 1,033 (5.8%) | 382 (1.4%) |
| MEDEA deprivation index, quintiles |  |  |  |  |  |
| First (least deprived) | 281,298 (13.5%) | 4,795 (12.4%) | 22,223 (10.9%) | 2,500 (14.1%) | 5,158 (18.3%) |
| Second | 276,440 (13.2%) | 5,284 (13.7%) | 27,256 (13.3%) | 2,602 (14.6%) | 3,960 (14.1%) |
| Third | 279,562 (13.4%) | 5,505 (14.3%) | 29,828 (14.6%) | 2,594 (14.6%) | 3,596 (12.8%) |
| Fourth | 293,611 (14.1%) | 5,753 (14.9%) | 32,163 (15.7%) | 2,461 (13.8%) | 3,393 (12.1%) |
| Fifth (most deprived) | 324,322 (15.5%) | 5,954 (15.4%) | 34,191 (16.7%) | 2,413 (13.6%) | 3,390 (12.1%) |
| Rural | 281,260 (13.5%) | 5,281 (13.7%) | 27,229 (13.3%) | 2,576 (14.8%) | 4,587 (16.3%) |
| Missing | 352,148 (16.9%) | 6,015 (15.6%) | 31,886 (15.6%) | 2,635 (14.5%) | 4,040 (14.4%) |
| Nationality |  |  |  |  |  |
| Spain | 1,631,936 (78.1%) | 34,099 (88.4%) | 178,179 (87.0%) | 16,343 (91.9%) | 26,746 (95.1%) |
| Other European countries | 106,782 (5.1%) | 1,258 (3.3%) | 7,166 (3.5%) | 469 (2.6%) | 428 (1.5%) |
| Americas | 149,109 (7.1%) | 1,945 (5.0%) | 10,204 (5.0%) | 625 (3.5%) | 574 (2.0%) |
| Africa | 124,013 (5.9%) | 937 (2.4%) | 7,175 (3.5%) | 227 (1.3%) | 304 (1.1%) |
| Asia and Oceania | 76,801 (3.7%) | 348 (0.9%) | 2,052 (1.0%) | 117 (0.7%) | 72 (0.3%) |
| Note: The Total column reflects the characteristics of all individuals upon cohort entry throughout the study period. In specific diagnosis columns, characteristics are determined at the time of diagnosis. | | | | | |

**Table S2. Annual IRs (per 100,000 persons at risk) of mental health disorders in young people in Catalonia, overall and by sex, 2008-2022**

|  |  | **2008** | **2009** | **2010** | **2011** | **2012** | **2013** | **2014** | **2015** | **2016** | **2017** | **2018** | **2019** | **2020** | **2021** | **2022** |
| --- | --- | --- | --- | --- | --- | --- | --- | --- | --- | --- | --- | --- | --- | --- | --- | --- |
| Depressive disorders | Overall | 193 | 242 | 238 | 208 | 217 | 292 | 319 | 295 | 279 | 285 | 317 | 328 | 287 | 461 | 472 |
|  | Girls | 252 | 314 | 309 | 276 | 280 | 378 | 404 | 376 | 347 | 362 | 401 | 414 | 383 | 646 | 647 |
|  | Boys | 137 | 174 | 171 | 143 | 157 | 211 | 239 | 218 | 214 | 213 | 238 | 248 | 199 | 291 | 310 |
| Anxiety disorders | Overall | 1089 | 1280 | 1258 | 1256 | 1318 | 1614 | 1743 | 1742 | 1713 | 1726 | 1859 | 2046 | 1743 | 2462 | 2537 |
|  | Girls | 1460 | 1687 | 1649 | 1637 | 1738 | 2114 | 2273 | 2263 | 2221 | 2247 | 2450 | 2684 | 2294 | 3431 | 3501 |
|  | Boys | 744 | 902 | 895 | 904 | 933 | 1155 | 1258 | 1268 | 1250 | 1252 | 1324 | 1475 | 1254 | 1608 | 1695 |
| Eating disorders | Overall | 76 | 102 | 102 | 95 | 120 | 160 | 151 | 139 | 113 | 102 | 110 | 114 | 131 | 264 | 249 |
|  | Girls | 136 | 181 | 180 | 173 | 217 | 295 | 271 | 246 | 198 | 169 | 192 | 199 | 241 | 506 | 465 |
|  | Boys | 19 | 27 | 29 | 22 | 30 | 33 | 39 | 39 | 33 | 38 | 34 | 34 | 30 | 42 | 52 |
| Attention deficit/  hyperactivity disorder | Overall | 132 | 196 | 233 | 246 | 276 | 291 | 241 | 205 | 190 | 203 | 197 | 201 | 138 | 258 | 306 |
|  | Girls | 56 | 91 | 113 | 133 | 158 | 155 | 145 | 123 | 116 | 108 | 109 | 110 | 88 | 177 | 212 |
|  | Boys | 205 | 297 | 350 | 356 | 390 | 423 | 335 | 286 | 262 | 296 | 284 | 289 | 187 | 336 | 397 |

**Table S3. Annual IRs (per 100,000 persons at risk) of mental health disorders in young people in Catalonia, by age groups, 2008-2022**

|  |  | **2008** | **2009** | **2010** | **2011** | **2012** | **2013** | **2014** | **2015** | **2016** | **2017** | **2018** | **2019** | **2020** | **2021** | **2022** |
| --- | --- | --- | --- | --- | --- | --- | --- | --- | --- | --- | --- | --- | --- | --- | --- | --- |
| Depressive disorders | 10-14 years | 68 | 116 | 106 | 96 | 98 | 200 | 257 | 245 | 240 | 245 | 250 | 254 | 246 | 419 | 372 |
|  | 15-18 years | 139 | 188 | 192 | 182 | 200 | 278 | 329 | 293 | 274 | 270 | 308 | 299 | 279 | 465 | 465 |
|  | 19-24 years | 319 | 362 | 365 | 311 | 324 | 380 | 367 | 341 | 317 | 332 | 385 | 415 | 329 | 497 | 563 |
| Anxiety disorders | 10-14 years | 397 | 602 | 676 | 654 | 723 | 935 | 1102 | 1104 | 1106 | 1105 | 1157 | 1235 | 1120 | 1632 | 1560 |
|  | 15-18 years | 811 | 1020 | 1028 | 1093 | 1227 | 1593 | 1780 | 1814 | 1749 | 1730 | 1872 | 2052 | 1708 | 2546 | 2485 |
|  | 19-24 years | 1802 | 1934 | 1857 | 1857 | 1890 | 2228 | 2309 | 2298 | 2273 | 2333 | 2544 | 2839 | 2361 | 3210 | 3516 |
| Eating disorders | 10-14 years | 65 | 94 | 98 | 95 | 125 | 176 | 157 | 151 | 135 | 106 | 122 | 122 | 137 | 298 | 307 |
|  | 15-18 years | 106 | 149 | 143 | 137 | 185 | 250 | 239 | 218 | 150 | 148 | 148 | 170 | 220 | 423 | 358 |
|  | 19-24 years | 65 | 81 | 81 | 69 | 78 | 91 | 90 | 78 | 69 | 67 | 74 | 68 | 66 | 127 | 126 |
| Attention deficit/  hyperactivity disorder | 10-14 years | 337 | 497 | 557 | 567 | 606 | 647 | 538 | 456 | 407 | 438 | 428 | 435 | 288 | 519 | 603 |
|  | 15-18 years | 78 | 133 | 172 | 185 | 238 | 223 | 171 | 133 | 140 | 133 | 120 | 131 | 98 | 204 | 237 |
|  | 19-24 years | 16 | 26 | 35 | 40 | 45 | 46 | 36 | 36 | 31 | 38 | 40 | 38 | 32 | 64 | 96 |

**Table S4. Annual IRs (per 100,000 persons at risk) of mental health disorders in young people in Catalonia, by deprivation status, 2008-2022**

|  |  | **2008** | **2009** | **2010** | **2011** | **2012** | **2013** | **2014** | **2015** | **2016** | **2017** | **2018** | **2019** | **2020** | **2021** | **2022** |
| --- | --- | --- | --- | --- | --- | --- | --- | --- | --- | --- | --- | --- | --- | --- | --- | --- |
| Depressive disorders | Q1 (least deprived) | 149 | 204 | 226 | 163 | 173 | 249 | 277 | 233 | 219 | 275 | 260 | 293 | 260 | 440 | 439 |
|  | Q2 | 162 | 250 | 216 | 167 | 221 | 282 | 315 | 288 | 297 | 256 | 295 | 330 | 289 | 483 | 491 |
|  | Q3 | 202 | 214 | 247 | 231 | 189 | 308 | 321 | 300 | 308 | 306 | 337 | 349 | 280 | 469 | 494 |
|  | Q4 | 199 | 264 | 260 | 235 | 231 | 307 | 343 | 311 | 275 | 304 | 349 | 341 | 305 | 490 | 469 |
|  | Q5 (most deprived) | 211 | 227 | 219 | 200 | 229 | 291 | 337 | 283 | 306 | 299 | 344 | 336 | 293 | 484 | 502 |
|  | Rural | 145 | 180 | 173 | 160 | 161 | 210 | 244 | 236 | 193 | 188 | 225 | 235 | 219 | 344 | 361 |
| Anxiety disorders | Q1 (least deprived) | 758 | 909 | 885 | 880 | 897 | 1177 | 1240 | 1328 | 1238 | 1248 | 1399 | 1615 | 1359 | 1988 | 1989 |
|  | Q2 | 982 | 1138 | 1094 | 1144 | 1260 | 1522 | 1626 | 1666 | 1651 | 1600 | 1837 | 1965 | 1687 | 2450 | 2478 |
|  | Q3 | 1059 | 1367 | 1319 | 1341 | 1395 | 1679 | 1856 | 1823 | 1824 | 1840 | 1901 | 2142 | 1827 | 2677 | 2736 |
|  | Q4 | 1236 | 1440 | 1379 | 1441 | 1491 | 1796 | 2012 | 1999 | 1931 | 1918 | 2065 | 2374 | 1939 | 2715 | 2850 |
|  | Q5 (most deprived) | 1261 | 1387 | 1443 | 1420 | 1481 | 1873 | 2009 | 1951 | 1921 | 2049 | 2130 | 2260 | 1979 | 2654 | 2844 |
|  | Rural | 757 | 861 | 834 | 816 | 895 | 1102 | 1212 | 1186 | 1179 | 1205 | 1337 | 1388 | 1257 | 1810 | 1892 |
| Eating disorders | Q1 (least deprived) | 74 | 98 | 102 | 91 | 122 | 164 | 141 | 149 | 108 | 91 | 108 | 110 | 138 | 267 | 244 |
|  | Q2 | 68 | 99 | 100 | 82 | 127 | 162 | 152 | 156 | 129 | 108 | 117 | 129 | 140 | 279 | 276 |
|  | Q3 | 69 | 112 | 91 | 108 | 129 | 156 | 156 | 144 | 118 | 101 | 130 | 121 | 142 | 289 | 260 |
|  | Q4 | 71 | 94 | 120 | 90 | 103 | 161 | 154 | 133 | 95 | 101 | 108 | 108 | 130 | 257 | 257 |
|  | Q5 (most deprived) | 72 | 94 | 92 | 84 | 132 | 152 | 143 | 111 | 116 | 101 | 85 | 83 | 112 | 236 | 224 |
|  | Rural | 60 | 69 | 74 | 70 | 83 | 122 | 115 | 105 | 85 | 74 | 81 | 101 | 103 | 231 | 207 |
| Attention deficit/  hyperactivity disorder | Q1 (least deprived) | 199 | 295 | 350 | 354 | 394 | 404 | 283 | 260 | 203 | 246 | 244 | 232 | 181 | 301 | 373 |
|  | Q2 | 155 | 227 | 233 | 267 | 285 | 296 | 278 | 214 | 174 | 188 | 196 | 182 | 137 | 247 | 293 |
|  | Q3 | 135 | 175 | 203 | 239 | 269 | 267 | 216 | 189 | 179 | 195 | 185 | 183 | 117 | 238 | 273 |
|  | Q4 | 105 | 165 | 192 | 206 | 220 | 205 | 197 | 174 | 160 | 174 | 164 | 191 | 121 | 253 | 288 |
|  | Q5 (most deprived) | 106 | 150 | 174 | 168 | 221 | 233 | 193 | 158 | 182 | 155 | 155 | 161 | 97 | 233 | 270 |
|  | Rural | 106 | 168 | 209 | 209 | 230 | 257 | 220 | 184 | 191 | 179 | 195 | 194 | 129 | 195 | 275 |

Note: Q designate the quintiles of the MEDEA index of socioeconomic deprivation, from Q1 (least deprivation) to Q5 (greatest deprivation). The MEDEA index does not apply to rural areas and its incidence rates are reported separately.

**Table S5. Annual IRs (per 100,000 persons at risk) of mental health disorders in young people in Catalonia, by nationality, 2008-2022**

|  |  | **2008** | **2009** | **2010** | **2011** | **2012** | **2013** | **2014** | **2015** | **2016** | **2017** | **2018** | **2019** | **2020** | **2021** | **2022** |
| --- | --- | --- | --- | --- | --- | --- | --- | --- | --- | --- | --- | --- | --- | --- | --- | --- |
| Depressive disorders | Spain | 205 | 257 | 252 | 215 | 227 | 306 | 337 | 304 | 286 | 289 | 319 | 332 | 292 | 469 | 478 |
|  | Rest of Europe | 148 | 195 | 199 | 197 | 187 | 241 | 250 | 303 | 277 | 353 | 381 | 342 | 295 | 399 | 431 |
|  | Americas | 130 | 219 | 201 | 239 | 219 | 259 | 273 | 308 | 302 | 343 | 424 | 481 | 389 | 683 | 719 |
|  | Africa | 104 | 117 | 141 | 118 | 127 | 202 | 164 | 186 | 184 | 185 | 217 | 204 | 184 | 292 | 267 |
|  | Asia and Oceania | 29 | 58 | 31 | 57 | 73 | 90 | 68 | 75 | 99 | 119 | 142 | 134 | 116 | 191 | 217 |
| Anxiety disorders | Spain | 1137 | 1341 | 1303 | 1308 | 1381 | 1675 | 1803 | 1790 | 1737 | 1743 | 1861 | 2034 | 1748 | 2486 | 2530 |
|  | Rest of Europe | 917 | 1129 | 1172 | 1081 | 1128 | 1457 | 1488 | 1646 | 1862 | 1915 | 2247 | 2255 | 1888 | 2427 | 2649 |
|  | Americas | 967 | 1079 | 1154 | 1195 | 1097 | 1336 | 1592 | 1730 | 1975 | 2174 | 2405 | 2899 | 2196 | 3336 | 4033 |
|  | Africa | 729 | 917 | 985 | 1001 | 1076 | 1401 | 1474 | 1532 | 1432 | 1492 | 1691 | 2109 | 1799 | 2141 | 1876 |
|  | Asia and Oceania | 250 | 325 | 487 | 352 | 462 | 547 | 660 | 539 | 731 | 642 | 766 | 788 | 631 | 998 | 1077 |
| Eating disorders | Spain | 81 | 111 | 110 | 100 | 130 | 175 | 162 | 147 | 120 | 105 | 112 | 122 | 143 | 291 | 269 |
|  | Rest of Europe | 57 | 60 | 67 | 81 | 83 | 72 | 110 | 147 | 79 | 113 | 116 | 77 | 99 | 177 | 215 |
|  | Americas | 59 | 87 | 100 | 110 | 117 | 116 | 87 | 82 | 71 | 105 | 150 | 96 | 115 | 142 | 225 |
|  | Africa | 28 | 32 | 24 | 41 | 37 | 34 | 52 | 62 | 42 | 31 | 58 | 39 | 23 | 79 | 69 |
|  | Asia and Oceania | 22 | 23 | 21 | 9 | 18 | 43 | 39 | 50 | 30 | 43 | 49 | 32 | 19 | 56 | 67 |
| Attention deficit/  hyperactivity disorder | Spain | 148 | 222 | 266 | 280 | 316 | 327 | 266 | 225 | 209 | 220 | 216 | 222 | 155 | 284 | 332 |
|  | Rest of Europe | 44 | 75 | 58 | 62 | 80 | 100 | 111 | 101 | 68 | 99 | 103 | 70 | 76 | 145 | 226 |
|  | Americas | 28 | 64 | 83 | 105 | 63 | 82 | 122 | 86 | 57 | 84 | 111 | 112 | 60 | 186 | 266 |
|  | Africa | 15 | 32 | 27 | 36 | 46 | 59 | 43 | 69 | 67 | 94 | 65 | 87 | 44 | 69 | 125 |
|  | Asia and Oceania | 22 | 12 | 10 | 19 | 14 | 24 | 19 | 15 | 10 | 38 | 9 | 16 | 4 | 52 | 53 |
